# Author Correction: Histone H2A Lys130 acetylation epigenetically regulates androgen production in prostate cancer

**DOI:** 10.1038/s41467-023-41810-9

**Published:** 2023-09-27

**Authors:** Thanh Nguyen, Dhivya Sridaran, Surbhi Chouhan, Cody Weimholt, Audrey Wilson, Jingqin Luo, Tiandao Li, John Koomen, Bin Fang, Nagireddy Putluri, Arun Sreekumar, Felix Y. Feng, Kiran Mahajan, Nupam P. Mahajan

**Affiliations:** 1 Department of Surgery, Cancer Research Building, Washington University in St Louis, 660 Euclid Ave., St Louis, MO 63110 USA; 2 Department of Urology, Cancer Research Building, Washington University in St Louis, 660 Euclid Ave., St Louis, MO 63110 USA; 3 Siteman Cancer Center, Cancer Research Building, Washington University in St Louis, 660 Euclid Ave., St Louis, MO 63110 USA; 4 Department of Pathology & Immunology, Cancer Research Building, Washington University in St Louis, 660 Euclid Ave., St Louis, MO 63110 USA; 5 Division of Public Health Sciences, Cancer Research Building, Washington University in St Louis, 660 Euclid Ave., St Louis, MO 63110 USA; 6 Bioinformatics Research Core, Center of Regenerative Medicine, Department of Developmental Biology, Washington University at St. Louis, St Louis, MO 63110 USA; 7 Molecular Oncology and Molecular Medicine, Moffitt Cancer Center, Tampa, FL 33612 USA; 8 Dan L Duncan Comprehensive Cancer Center, Baylor College of Medicine, Houston, TX 77030 USA; 9 Helen Diller Family Comprehensive Cancer Center, University of California, San Francisco, CA 94158 USA; 10 Present Address: Section of Gastroenterology & Hepatology, Department of Medicine, Baylor College of Medicine, Houston, TX 77030 USA

**Keywords:** Prostate, Gene silencing, Prostate cancer

Correction to: *Nature Communications* 10.1038/s41467-023-38887-7, published online 09 June 2023

This Article omits a declaration from the Competing Interests statement, which should have included the following: ‘A patent “Inhibitors of ACK1/TNK2 Tyrosine Kinase” (patent no. 9,850,216; 10,017,478 and 10,336,734) covers (R)-9b compound. NPM and KM are named as inventors. These patents have been licensed by TechnoGenesys Inc. KM and NPM are co-founders of TechnoGenesys Inc., own stocks, and serve as consultants for TechnoGenesys Inc. The other authors declare no potential conflicts of interest.’

These errors have now been corrected in either the PDF or HTML versions of the article.

