# Supplementary material for: Histone H2A Lys130 acetylation epigenetically regulates androgen production in prostate cancer
Source: Nat Commun. 2023 Jun 9;14:3357. doi: 10.1038/s41467-023-38887-7 (PMC10256812; doi:10.1038/s41467-023-38887-7)
Supplement: Supplementary file 1 — Supplementary Information [file 41467_2023_38887_MOESM1_ESM.pdf]

SUPPLEMENTARY INFORMATION

Histone H2A Lys130 Acetylation Epigenetically Regulates Androgen Production in Prostate Cancer

Thanh Nguyen[1,2 #], Dhivya Sridaran[1,2 #], Surbhi Chouhan[1,2], Cody Weimholt[3,4], Audrey Wilson[1,2], Jingqin Luo[5], Tiandao Li[6], John Koomen[7], Bin Fang[7], Nagireddy Putluri[8], Arun Sreekumar[8], Felix Y. Feng[9], Kiran Mahajan[1,2], Nupam P. Mahajan[1,2,3*]

Supplementary Fig. S1

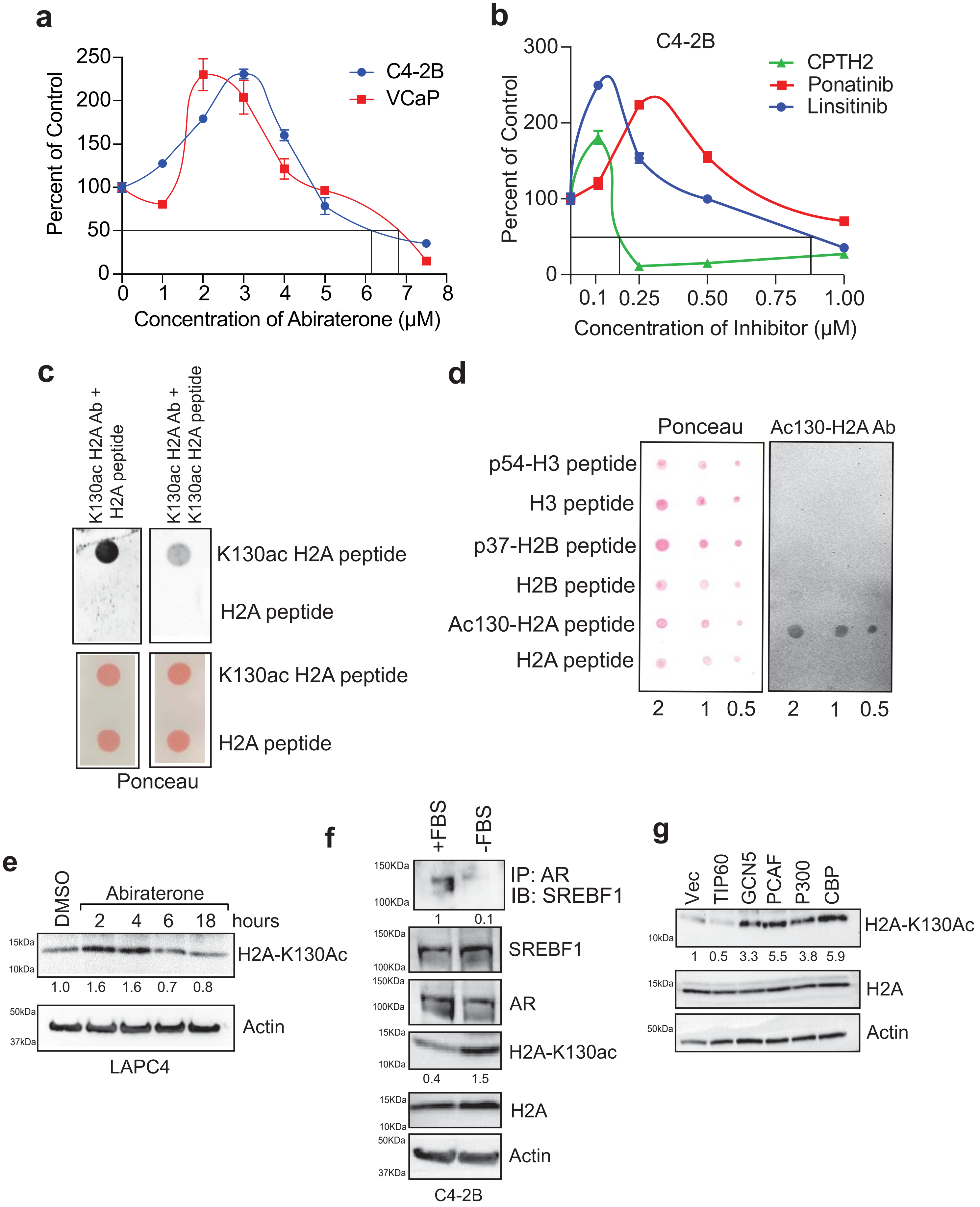

Supplementary Fig. S1: Identification of H2A Lys130-acetylation and generation of H2A-K130ac antibodies.

a C4-2B and VCaP cells were treated with increasing concentration of abiratetone and number of viable cells were counted by trypan blue exclusion assay. **b** C4-2B cells were treated with increasing concentration of CPTH2, Ponatinib, or Linsitinib for 96 hours and number of viable cells were counted by trypan blue exclusion assay. **c** Acetylated-K130 histone H2A peptide and corresponding non-acetylated peptides were immunoblotted with H2A-K130ac antibody (left panel) or H2A-K130ac antibody incubated with acetylate-K130 histone H2A peptide (right panel). Lower blot is Ponceau S stained. **d** Acetylated-K130 histone H2A peptide and many other histone-desrived peptides were immunoblotted with H2A-K130ac antibody (right panel) or Ponceau S stained (left panel). **e** LAPC4 cells were stimulated with abiraterone (7.5 μM) for 2, 4, 6 or 18 hours in -FBS media. Lysates were immunoprecipitated with H2A-K130ac antibody, followed by immunoblotting with H2A antibody. Lysates were also subjected to immunoblotting with H2A and Actin antibodies. **f** C4-2B cells were incubated + FBS and -FBS conditions for 40 hours. Lysates were immunoprecipitated with AR antibody followed by immunoblotting with SREBF1 antibody. Lysates were also immunoprecipitated with H2A-K130ac antibody, followed by immunoblotting with H2A antibody. Immunoblotting with SREBF1, AR, H2A and Actin antibodies were done. **g** HEK293T cells were transfected with pcDNA3.1 vector, TIP60, GCN5, PCAF, P300, or CBP acetyl transferases. Lysates were immunoprecipitated with K130ac-H2A antibody, followed by immunoblotting with H2A antibody. Representative images are as shown (n = 3 biologically independent experiments). Source data are provided as a Source Data file.

Supplementary Fig. S2

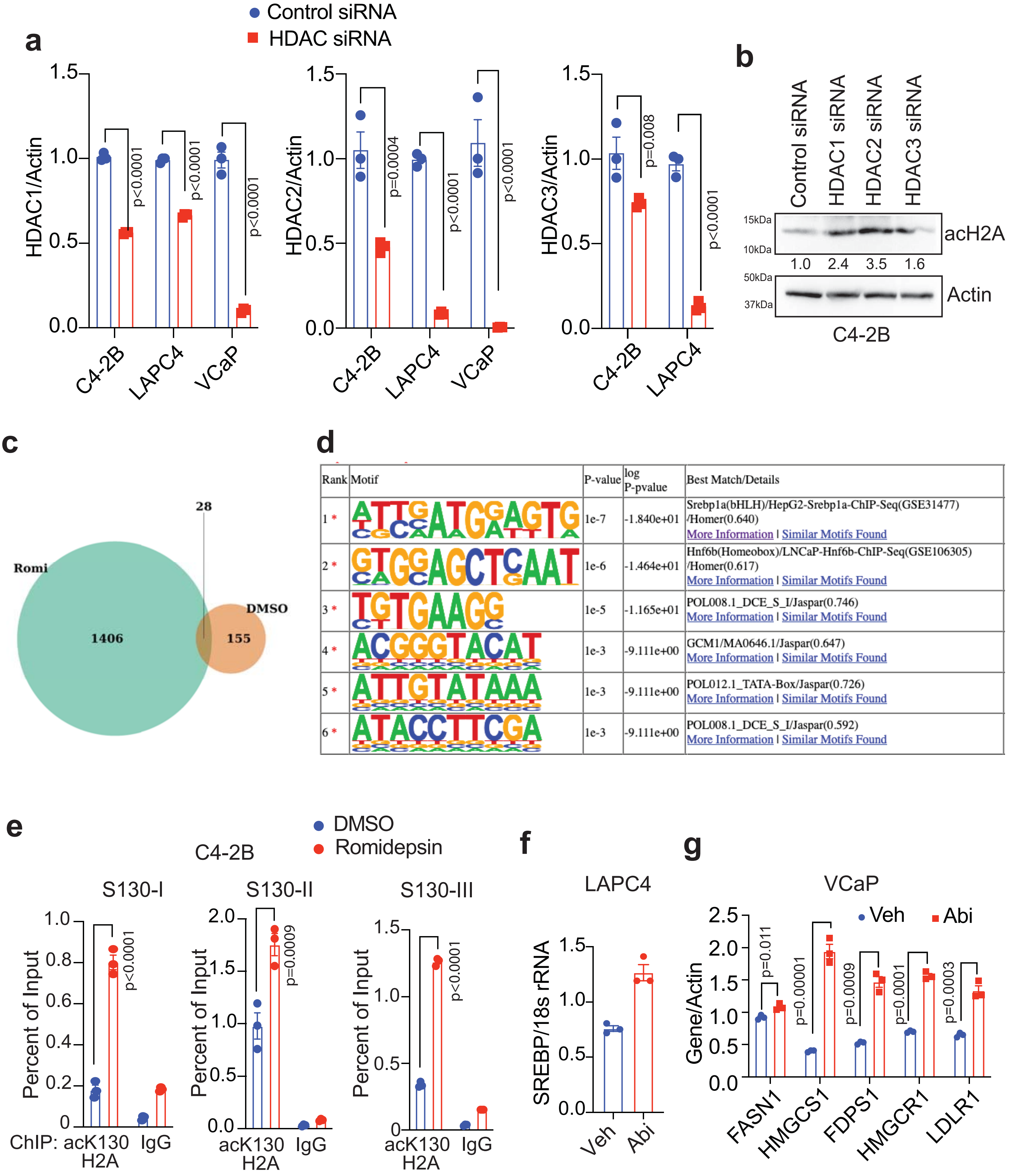

Supplementary Fig. S2

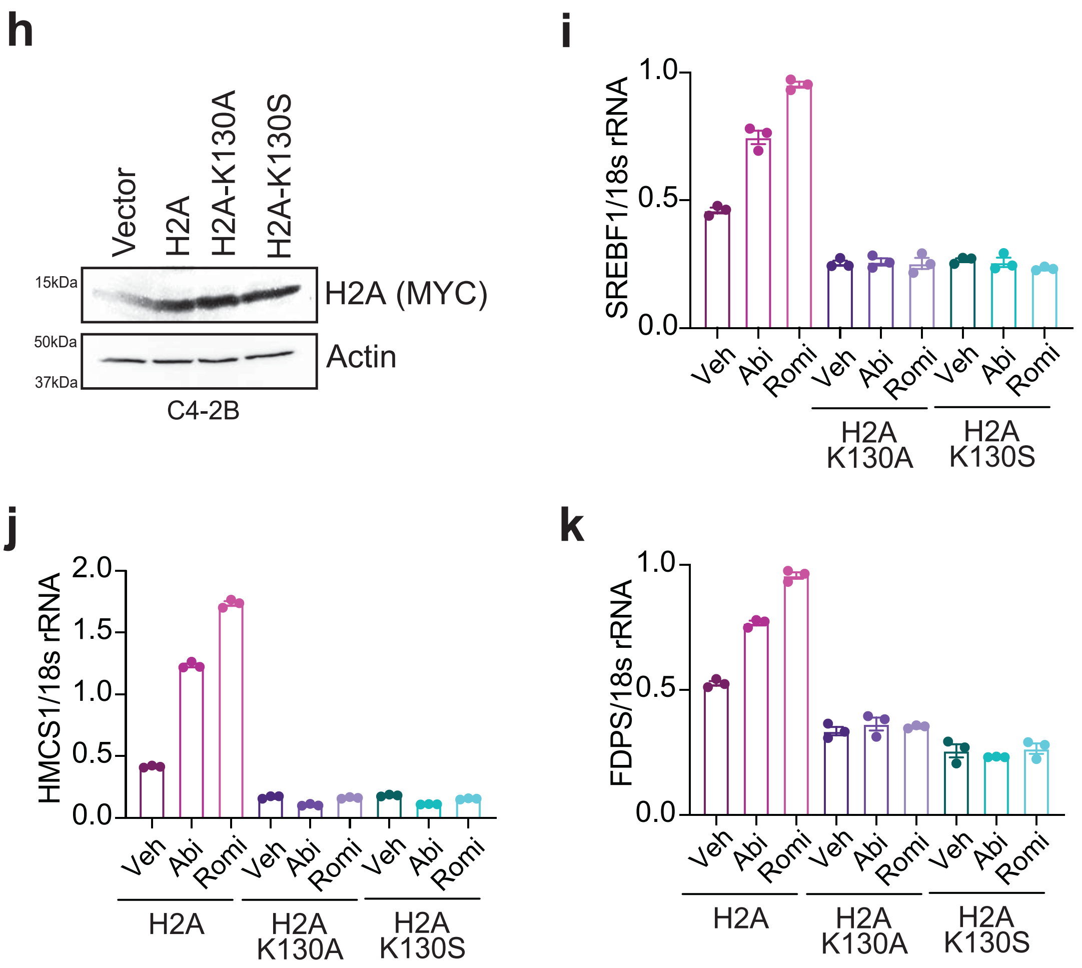

Supplementary Fig. S2: Identification of HATs and HDACs for H2A-K130ac epigenetic marks.

a C4-2B, VCaP and LAPC4 cells were transfected with HDAC1, 2 and 3 siRNAs, followed by qRT-PCR to assess levels of these HDACs mRNA levels. **b** C4-2B cells were transfected with HDAC1, 2 and 3 siRNAs, followed by immunoprecipitation with H2A-K130ac antibody, and immunoblotting with H2A antibody. Lysates were also subjected to immunoblotting with Actin antibodies. **c** Venn diagram of the unique and overlapping peaks identified in the VCaP cells treated with DMSO and Romidepsin (0.5 μM, 18h), followed by ChIP-seq using H2A-K130ac antibody. Default 5% intervals for motif finding was used. **d** H2A-K130ac ChIP-sequencing data was searched for the de novo transcription-factor-binding motif (analysis was performed using HOMER). The significantly enriched motifs and associated p values are shown. Motif enrichment is calculated using either the cumulative hypergeometric or cumulative binomial distributions. **e** C4-2B cells were treated with vehicle or Romidepsin (1μM, 18h) and ChIP was performed using H2A-K130ac antibody or IgG, followed by qPCR using primers corresponding to S130-I/II/III. **f** RNA isolated from LAPC4 cells treated with vehicle or abiraterone (7.5 μM, 18h) and was subjected to qRT-PCR with *SREBF1* and 18s rRNA primers. **g** RNA isolated from VCaP cells treated with vehicle or abiraterone (7.5 μM, 18h) and qRT-PCR was done with indicated primers. **h** C4-2B cells were infected with retroviral constructs expressing MYC- tagged wildtype H2A or mutants K130A-H2A, K130S-H2A. The transfections were confirmed by immunoblotting of the lysates with MYC antibody. **i-k** C4-2B cells were infected with retroviral constructs expressing wildtype H2A or mutants K130A-H2A, K130S-H2A followed by treatment with vehicle or abiraterone (7.5 μM) or Romidepsin (0.5 μM) for 18h. RNA isolated and was subjected to qRT-PCR with indicated primers. For **a**, **e**, and **g,** The experiments were performed in triplicates (n=3), and the experiments were repeated thrice independently with similar results; a representative dataset is shown. For **f, i, j** and **k** The experiments were performed in triplicates (n=3), and the experiments were repeated twice independently with similar results; a representative dataset is shown. Data are represented as mean ± SEM. For **a**, **e-g**, p values were determined by unpaired two-tailed Student's *t*-test. Source data are provided as a Source Data file.

Supplementary Fig. S3

a

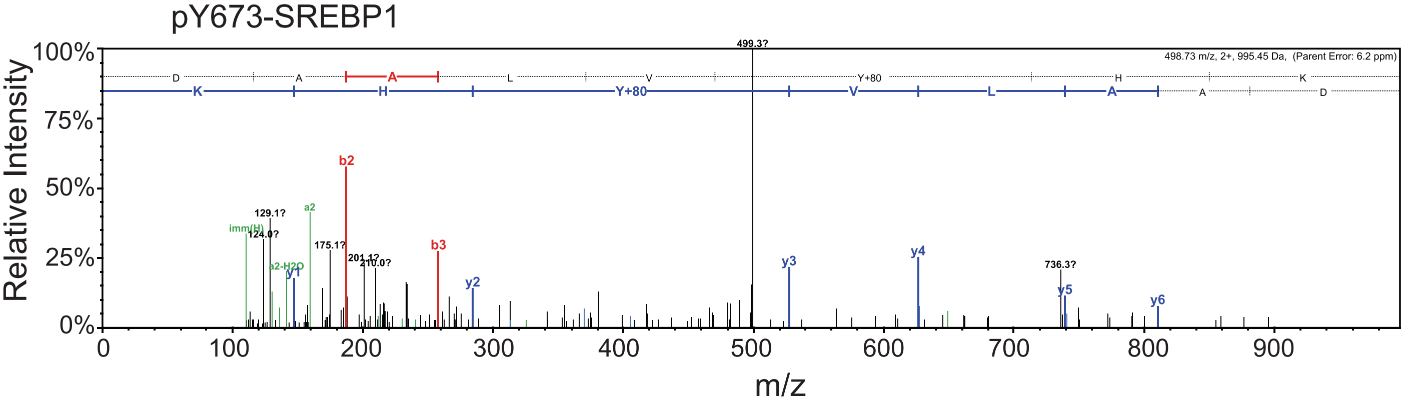

b

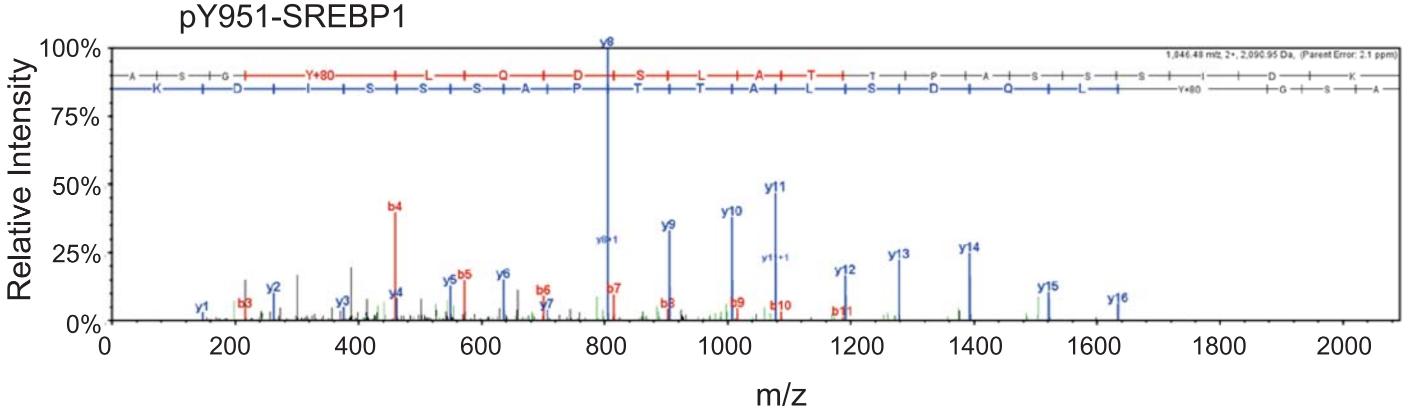

c

```
                    673
661 DASASARDAALVYHKLHQLH SREBP-1A Homo sapiens
637 DASASARDAALVYHKLHQLH SREBP-1C Homo sapiens
650 DARASARDAAVVYHKLHQLH Mus musculus (mouse)
614 EATTSARDAALVYHKLHQLH Xenopus laevis(Frog)
576 RNLRLPKRLRVVYFLARTRL Oryzias latipes(Fish)

                951
941 SLTICEKASGYLQDSLATTP SREBP-1A Homo sapiens
917 SLTICEKASGYLQDSLATTP SREBP-1C Homo sapiens
928 SLAICEKASGYLRDSLASTP Mus musculus(Mouse)
890 SFYHCEKASAFLWNSLNISS Xenopus laevis(Frog)
761 RPTLIPKISADLQTDLTLYK Oryzias latipes(Fish)
```

Supplementary Fig. S3: Identification of Tyr673- & Tyr951-Phosphorylation in SREBP1.

a The intact peptide (MS2) was detected with m/z of 498.73, which represents a ppm error of 6.2. The MS/MS spectrum was matched to peptide DAALVYHK with tyrosine 673 phosphorylated. The identification was made by both Mascot and Scaffold with a Mascot ion score of 14.6. **b** The intact peptide (MS3) was detected with m/z of 1,046.48, which represents a ppm error of 1.1. The MS/MS spectrum was matched to peptide ASGYLQDSLATTPASSSIDK with tyrosine 951 phosphorylated. The identification was made by Mascot ion score of 37.6. **c** Evolutionary conservation of Tyr673 and Tyr951 in SREBP1.

Supplementary Fig. S4

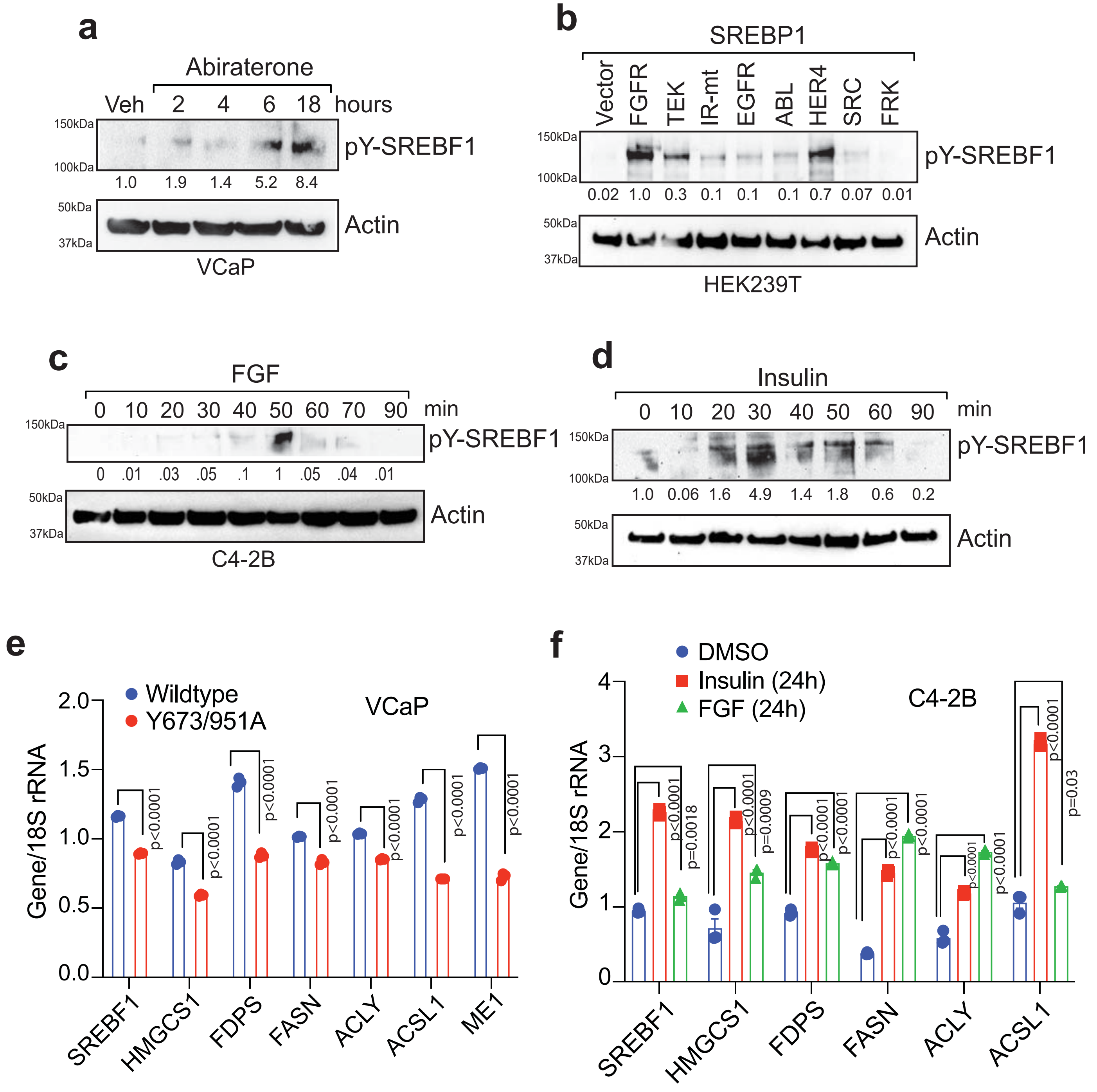

Supplementary Fig. S4: SREBP1 Phosphorylation augments SREBP1 and its target gene expression.

a Serum-starved VCaP cells were stimulated with abiraterone acetate (7.5 μM) for 2, 4, 6 or 18 hours. Lysates were immunoprecipitated with SREBP1 antibodies, followed by immunoblotting with pTyr antibody. **b** HEK239T cells were co-transfected with SREBP1 and various tyrosine kinases (FGFR, TEK, IR, EGFR, ABL, HER4, SRC, or FRK) expressing constructs. Lysates were immunoprecipitated with pTyr antibodies, followed by immunoblotting with SREBP1 antibody. **c** Serum-starved C4-2B cells were stimulated with FGF (10 nM) for indicated time. Lysates were immunoprecipitated with pTyr antibodies, followed by immunoblotting with SREBP1 antibody. **d** Serum-starved C4-2B cells were stimulated with insulin (10 nM) for indicated time. Lysates were immunoprecipitated with pTyr mouse mAb, followed by immunoblotting with SREBP1 antibody. **e** Serum-starved VCaP cells were iterated with SREBP1 or Y703/981A double mutant expressing retroviral constructs. Cells were treated with abiraterone (7.5μM) for 4 hours, followed by qRT-PCR with actin, SREBP1, and targeted-gene primers (HMGCS1, FDPS, FASN, ACLY, ACSL1 and ME1). **f** Serum-starved C4-2B cells were stimulated with Insulin or FGF (10 nM) for 24 hours. Total RNA was isolated, followed by qRT-PCR with actin, SREBP1, and targeted-gene primers (HMGCS1, FDPS, FASN, ACLY, and ACSL1). For **e** and **f** The experiments were performed in triplicates (n=3), and the experiments were repeated thrice independently with similar results; a representative dataset is shown. For **e** and **f**, data are represented as mean ± SEM. For **e**, p values were determined by unpaired two-tailed Student's *t*-test. For **f**, p values were determined by one-way ANOVA. Source data are provided as a Source Data file.

Supplementary Fig. S5

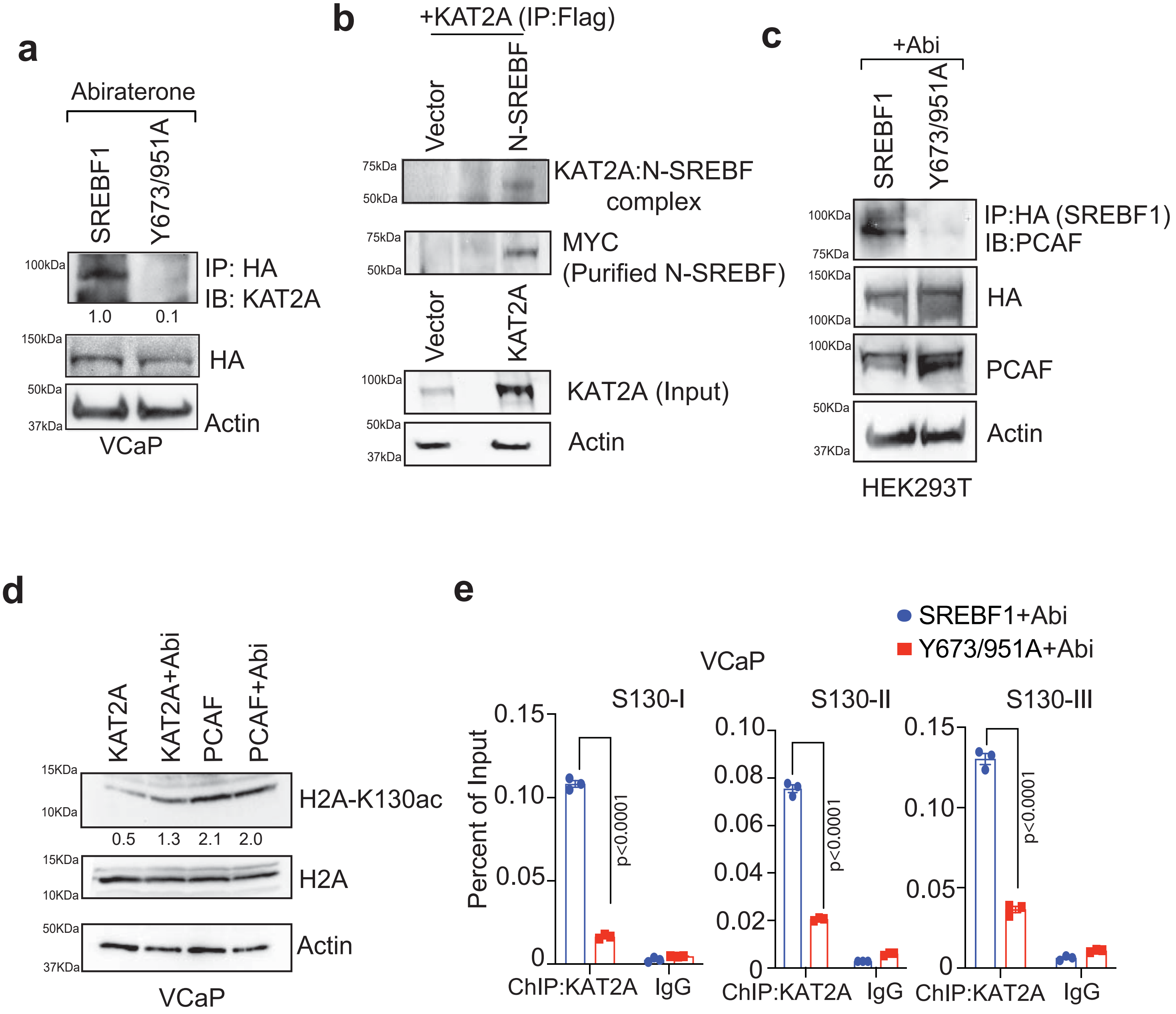

Supplementary Fig. S5: SREBP1 recruits KAT2A upon its nuclear translocation in Phosphorylation dependent manner

a VCaP cells were infected with HA-tagged SREBP1 or Y673/951A double mutant expressing retroviral constructs and treated with abiraterone (7.5μM). Lysates were immunoprecipitated with HA antibodies, followed by immunoblotting with KAT2A antibody. **b** HEK293T cells were transfected with vector or FLAG-tagged KAT2A or His and MYC-tagged N-SREBP1. Lysates were made. N-SREBP1 was purified using Ni-NTA (His beads). Ex vivo binding and complex formation was assessed by incubating purified N-SREBP1with KAT2A purified using FLAG beads followed by immunoblotting with MYC (N-SREBF1). **c** HEK293T cells were infected with HA-tagged SREBF1 or Y673/951A double-mutant expressing constructs. Lysates were immunoprecipitated with anti-HA affinity gel followed by immunoblotting with PCAF antibody. **d** VCaP cell were transfected with KAT2A or PCAF and treated with vehicle or abiraterone (7.5μM). Lysates were immunoprecipitated with H2A-K130ac antibody, followed by immunoblotting with H2A antibody. **e** VCaP cells were infected with SREBP1 or Y673/951A double mutant expressing retroviral constructs. Cells were treated with abiraterone (7.5μM) for 4 hours, followed by ChIP with KAT2A (or IgG) antibodies. PCR was performed using S130-I/II/III primers. For **e** The experiments were performed in triplicates (n=3), the experiment was repeated thrice independently with similar results; a representative dataset is shown. For **e**, data are represented as mean ± SEM and p values were determined by unpaired two-tailed Student's *t*-test. Source data are provided as a Source Data file.

Supplementary Fig. S6

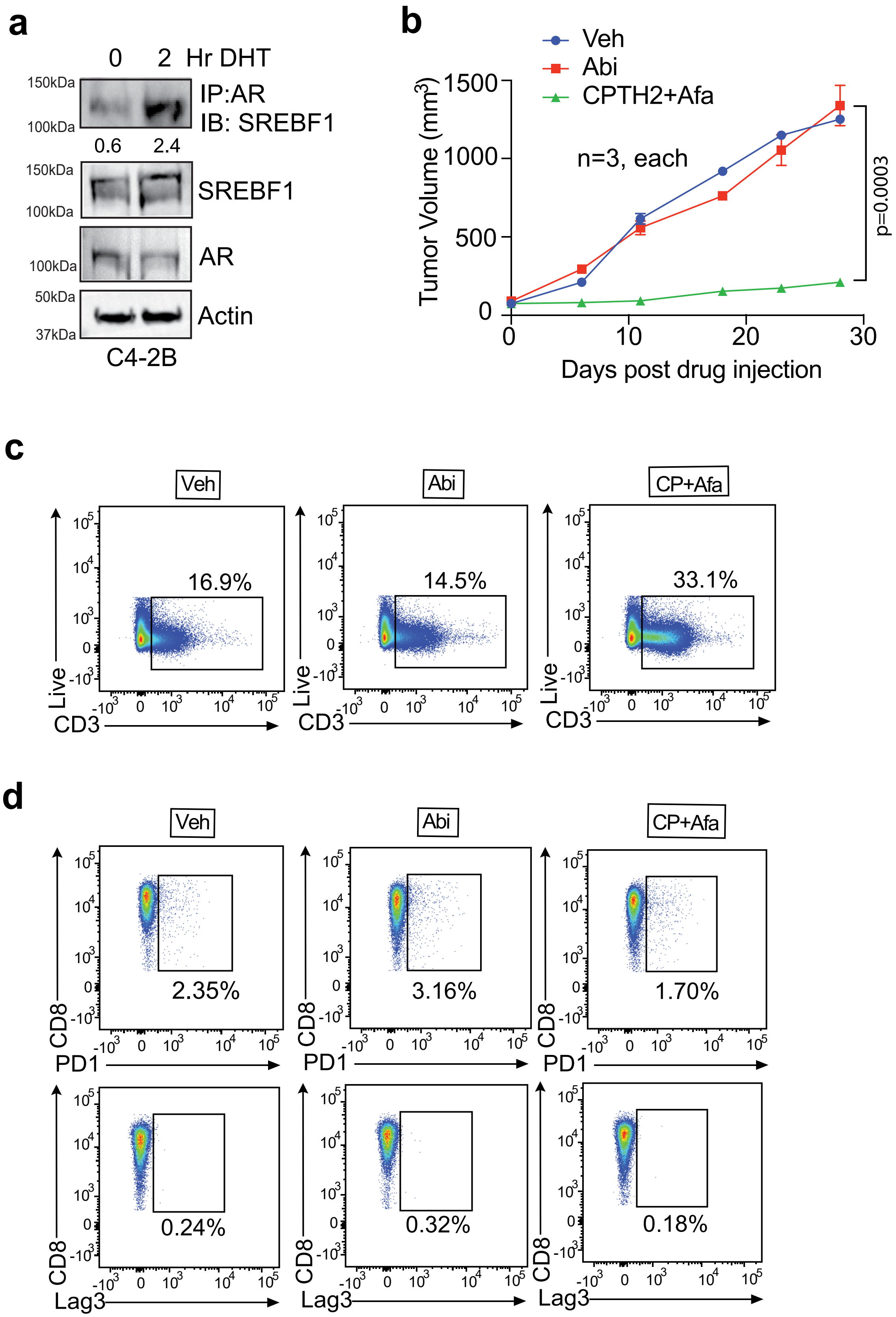

Supplementary Fig. S6

e

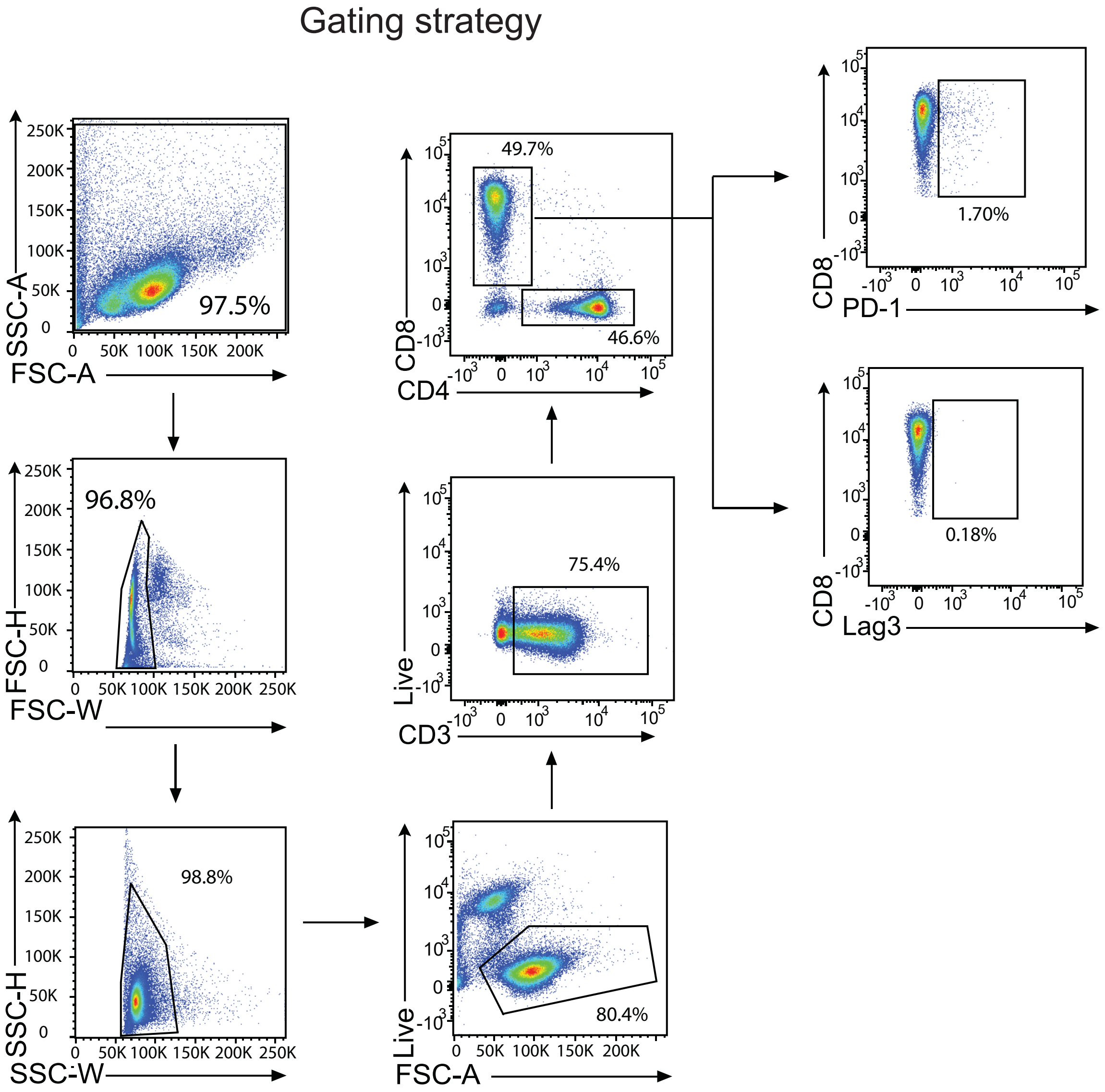

Supplementary Fig. S6: SREBP1-AR complex senses the androgen deficiency.

a C4-2B cells were DHT treated for indicated time. Lysates were immunoprecipitated with AR antibody, followed by immunoblotting with SREBP1 antibody. **b** TRAMP-C2 cells were implanted subcutaneously in C57BL/6 mice. When tumors became palpable, mice were orally gavaged with abiraterone (96 mg/kg of body weight) or CPTH2+Erdafitinib (12mg/kg of body weight) for five days a week for 4 weeks (n=3 mice in each arm). Tumor volumes were measured with calipers. **c** A representative flow cytometric analysis of CD3 gated population isolated from tumor draining lymph nodes of mice gavaged orally with vehicle, abiraterone or CPTH2 and Afatinib. **d** A representative flow cytometric analysis of exhaustion markers - PD1 and Lag3 on CD8 gated population isolated from tumor draining lymph nodes of mice gavaged orally with vehicle, abiraterone or CPTH2 and Afatinib. **e** A representative gating strategy of the flow cytometric analysis of CD3, CD8 and exhaustion markers. For **a**, the experiment was performed in triplicates and two biological replicates. For **b**, data are represented as mean ± SEM and p values were determined by unpaired two-tailed Student's *t*-test. Source data are provided as a Source Data file.

Figure S7

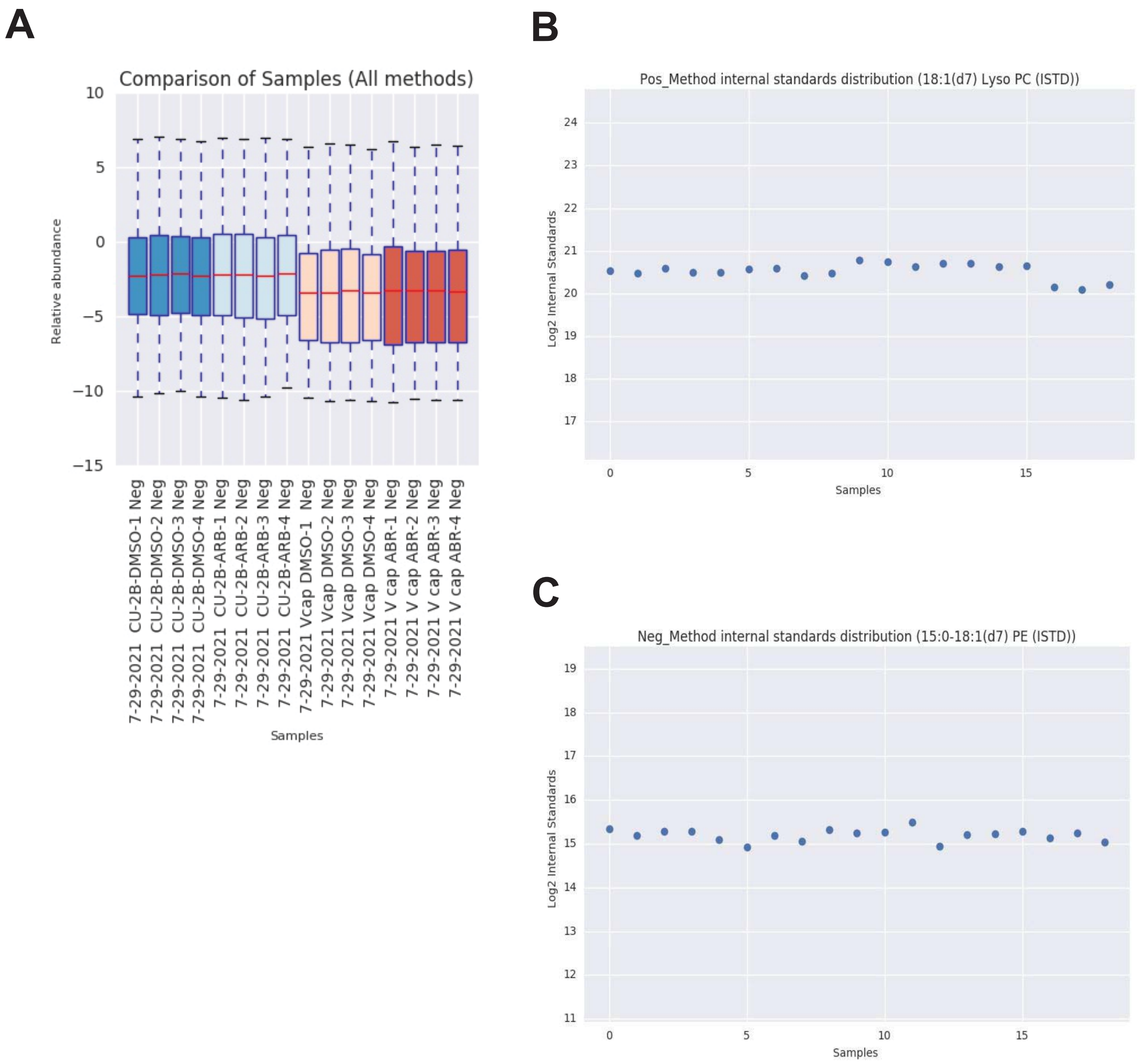
A
Comparison of Samples (All methods)
Relative abundance
Samples
7-29-2021 CU-2B-DMSO-1 Neg
7-29-2021 CU-2B-DMSO-2 Neg
7-29-2021 CU-2B-DMSO-3 Neg
7-29-2021 CU-2B-DMSO-4 Neg
7-29-2021 CU-2B-ARB-1 Neg
7-29-2021 CU-2B-ARB-2 Neg
7-29-2021 CU-2B-ARB-3 Neg
7-29-2021 CU-2B-ARB-4 Neg
7-29-2021 Vcap DMSO-1 Neg
7-29-2021 Vcap DMSO-2 Neg
7-29-2021 Vcap DMSO-3 Neg
7-29-2021 Vcap DMSO-4 Neg
7-29-2021 V cap ABR-1 Neg
7-29-2021 V cap ABR-2 Neg
7-29-2021 V cap ABR-3 Neg
7-29-2021 V cap ABR-4 Neg
B
Pos_Method internal standards distribution (18:1(d7) Lyso PC (ISTD))
Log2 Internal Standards
Samples
C
Neg_Method internal standards distribution (15:0-18:1(d7) PE (ISTD))
Log2 Internal Standards
Samples

Supplementary Fig. S7: Quality control data for the lipidomics profiling mass spectrometry platform.

a Distribution of lipids detected across all samples from both positive and negative mode (log transformed data). Each box has lines at the lower quartile (25%), median (50%), and upper quartile values (75%). Whiskers extend from each end of the box to the most extreme values within 1.5 times the interquartile range from the ends of the box. **b** and **c** Distribution of isotopic labelled internal standards which were spiked into the biological samples measured by (**b**) ESI-positive mode, (**c**) ESI-Negative mode. n=4.

Figure S8

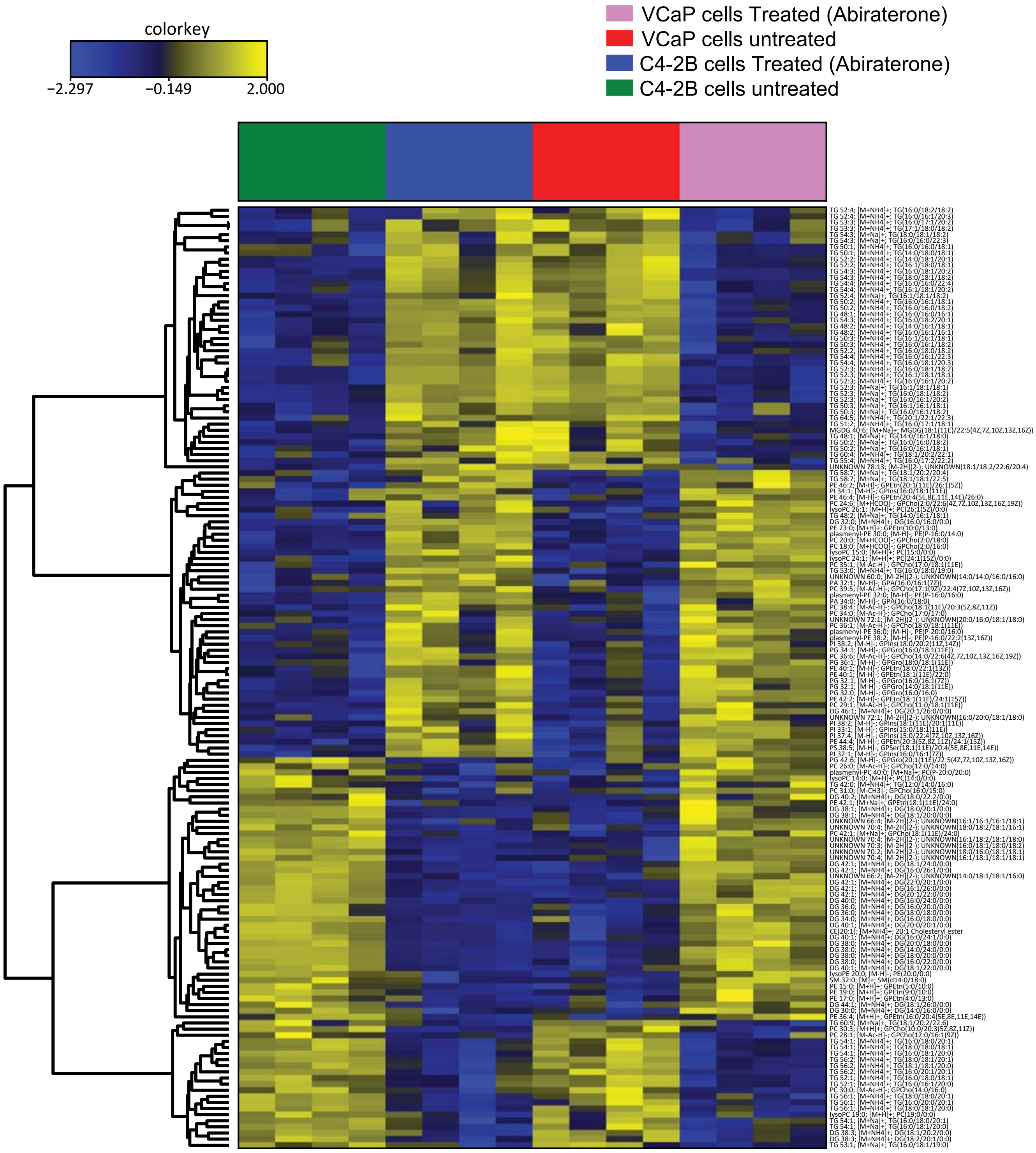
colorkey
−2.297
−0.149
2.000
VCaP cells Treated (Abiraterone)
VCaP cells untreated
C4-2B cells Treated (Abiraterone)
C4-2B cells untreated

Supplementary Fig. S8. Global lipidomics analysis of abiraterone-resistant CRPCs

Heat map showing significantly altered lipids (stratified by fatty acid chain length: degree of saturation) in C4-2B and VCaP cells untreated or treated with abiraterone. Shades of yellow and blue represent up and down regulated lipids, respectively (see color key).

Figure S9

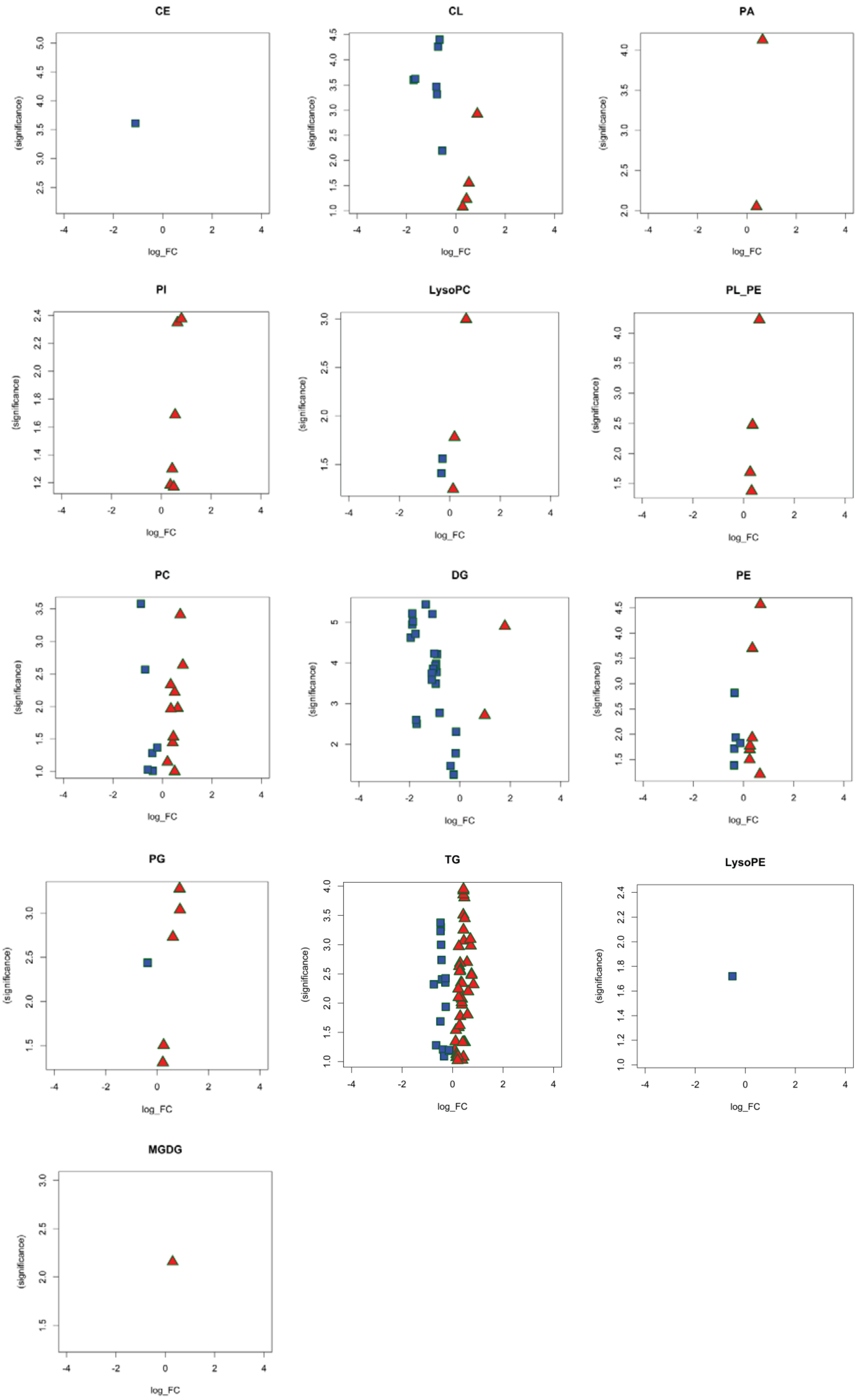
CE
CL
PA
PI
LysoPC
PL_PE
PC
DG
PE
PG
TG
LysoPE
MGDG
(significance)
log_FC

Supplementary Fig. S9. Stratification of the altered lipids within each class

Plots showing various altered lipids within each class stratified by significance Lipid classes include CE: Cholesteryl Esters, CL: cardiolipin, PA: phosphatidic acid, PI: Phosphatidyl inositol, LysoPC: Lyso phosphatidyl choline, PL-PE: Plasmenyl Phosphatidyl Ethanolamine, PC: Phosphatidyl choline, DG: Diglycerides, PE: Phosphatidyl ethanolamine, PG: Phosphatidyl Glycerol, TG: Triglycerides, LysoPE: Lyso Phosphatidyl Ethanolamine, MGDG: Monogalactosyldiacylglycerol.

Figure S10

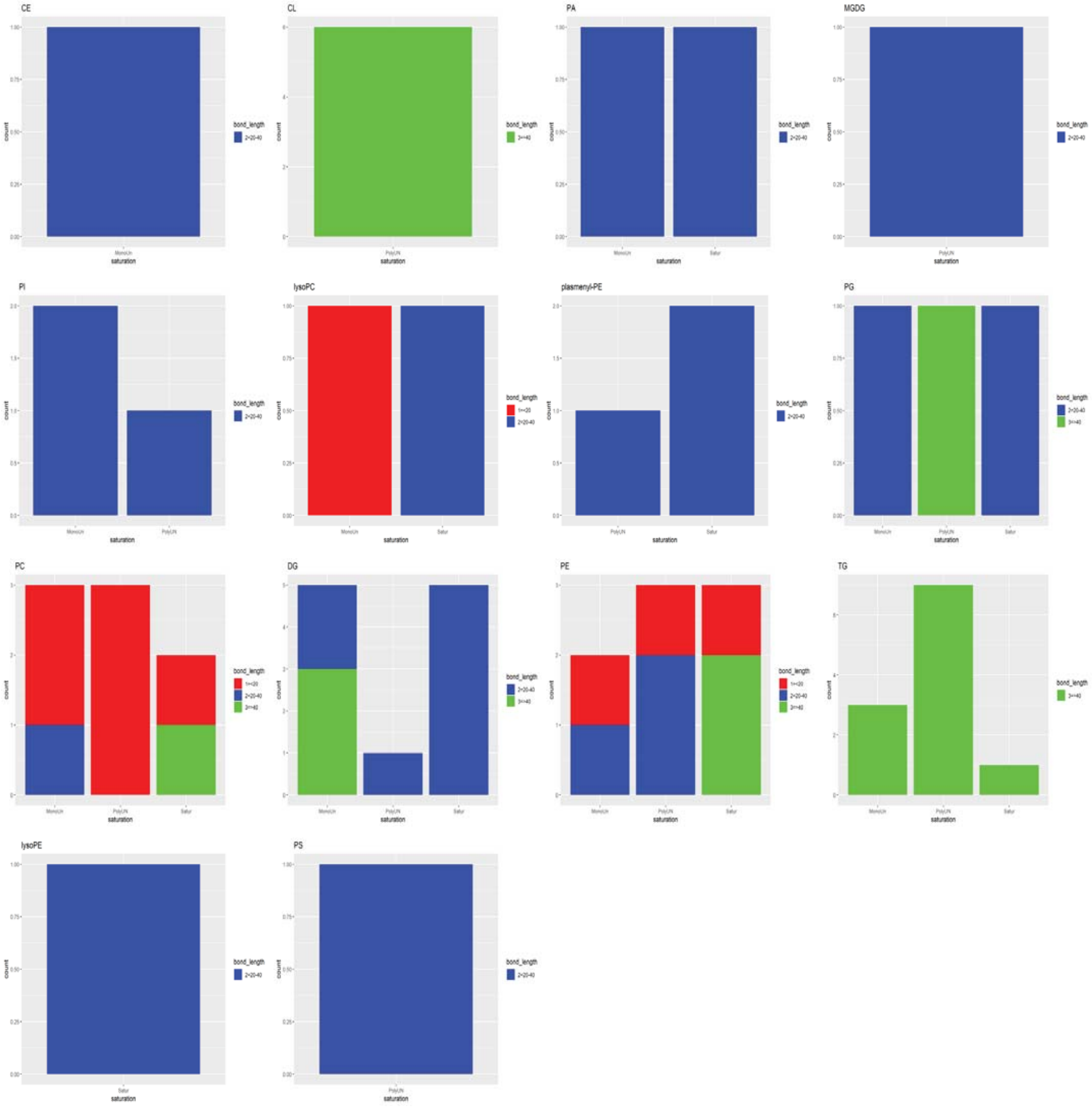
CE
CL
PA
MGDG
PI
lysoPC
plasmenyl-PE
PG
PC
DG
PE
TG
lysoPE
PS
count
saturation
bond_length

Supplementary Fig. S10. Heat map showing altered lipids stratified by bond length.

Lipid classes include Lipid classes include CE: Cholesteryl Esters, CL: cardiolipin, PA: phosphatidic acid, PI: Phosphatidyl inositol, LysoPC: Lyso phosphatidyl choline, PL-PE: Plasmenyl Phosphatidyl Ethanolamine, PC: Phosphatidyl choline, DG: Diglycerides, PE: Phosphatidyl ethanolamine, PG: Phosphatidyl Glycerol, TG: Triglycerides, LysoPE: Lyso Phosphatidyl Ethanolamine, MGDG: Monogalactosyldiacylglycerol..
