# Supplementary material for: Histone H2A Lys130 acetylation epigenetically regulates androgen production in prostate cancer
Source: Nat Commun. 2023 Jun 9;14:3357. doi: 10.1038/s41467-023-38887-7 (PMC10256812; doi:10.1038/s41467-023-38887-7)
Supplement: Supplementary file 3 — Description of Additional Supplementary Files [file 41467_2023_38887_MOESM3_ESM.pdf]

Description of Additional Supplementary Files

File Name: Supplementary Data 1
Description: VCaP ChIP-sequencing with ac130-H2A antibodies

File Name: Supplementary Data 2
Description: Metabolites identified in C4-2B cells treated with abiraterone

File Name: Supplementary Data 3
Description: Metabolites identified in VCaP cells treated with abiraterone

File Name: Supplementary Data 4
Description: Statistical analysis of Metabolite profiling

File Name: Supplementary Data 5
Description: Normalized metabolite profile

File Name: Supplementary Data 6
Description: Combined report of the metabolite profile

File Name: Supplementary Data 7
Description: List of primers
