# Supplementary material for: Histone H2A Lys130 acetylation epigenetically regulates androgen production in prostate cancer
Source: Nat Commun. 2023 Jun 9;14:3357. doi: 10.1038/s41467-023-38887-7 (PMC10256812; doi:10.1038/s41467-023-38887-7)
Supplement: Supplementary file 10 — Supplementary Data 7 [file 41467_2023_38887_MOESM10_ESM.pdf]

Supplementary Data 7. List of Primers

**ChIP PCR Primer**	
S130-1_FP: AAGTGCAATCCATGGCTCCG	This paper
S130-1_RP: AACGGCTTCAAAAATCCGCC	This paper
S130-2_FP: ATGAGGTTCCAGAGGAGGCT	This paper
S130-2_RP: ATCCACAGGGAGACTTTGCC	This paper
S130-3_FP: AGAGACCAGGGGACTGAGAC	This paper
S130-3_RP: CTTACAGCCAAGCCTCTCCC	This paper
**qRT-PCR Primers:**	
Human SREBF1 FP & RP	OriGene Technologies
Human HMGCS1 FP & RP	OriGene Technologies
Human FDPS1 FP & RP	OriGene Technologies
Human FASN1 FP & RP	OriGene Technologies
Human ELOVL6 FP & RP	OriGene Technologies
Human HMGCR1 FP & RP	OriGene Technologies
Human LDLR1 FP & RP	OriGene Technologies
Human ACLY FP & RP	OriGene Technologies
Human ACSL1 FP & RP	OriGene Technologies
Mouse Srebf1 FP & RP	OriGene Technologies
Mouse Fasn1 FP & RP	OriGene Technologies
Mouse Hmgcs1 FP & RP	OriGene Technologies
Mouse Fdps FP & RP	OriGene Technologies
