# Supplementary material for: Histone H2A Lys130 acetylation epigenetically regulates androgen production in prostate cancer
Source: Nat Commun. 2023 Jun 9;14:3357. doi: 10.1038/s41467-023-38887-7 (PMC10256812; doi:10.1038/s41467-023-38887-7)
Supplement: Supplementary file 11 — Reporting Summary [file 41467_2023_38887_MOESM11_ESM.pdf]

nature portfolio

Corresponding author(s): Nupam Mahajan

Last updated by author(s): May 2, 2023

Reporting Summary

Nature Portfolio wishes to improve the reproducibility of the work that we publish. This form provides structure for consistency and transparency in reporting. For further information on Nature Portfolio policies, see our Editorial Policies and the Editorial Policy Checklist.

Statistics

For all statistical analyses, confirm that the following items are present in the figure legend, table legend, main text, or Methods section.

n/a	Confirmed	
☐	☒	The exact sample size (*n*) for each experimental group/condition, given as a discrete number and unit of measurement
☐	☒	A statement on whether measurements were taken from distinct samples or whether the same sample was measured repeatedly
☐	☒	The statistical test(s) used AND whether they are one- or two-sided<br>*Only common tests should be described solely by name; describe more complex techniques in the Methods section.*
☐	☒	A description of all covariates tested
☐	☒	A description of any assumptions or corrections, such as tests of normality and adjustment for multiple comparisons
☐	☒	A full description of the statistical parameters including central tendency (e.g. means) or other basic estimates (e.g. regression coefficient) AND variation (e.g. standard deviation) or associated estimates of uncertainty (e.g. confidence intervals)
☐	☒	For null hypothesis testing, the test statistic (e.g. *F*, *t*, *r*) with confidence intervals, effect sizes, degrees of freedom and *P* value noted<br>*Give P values as exact values whenever suitable.*
☒	☐	For Bayesian analysis, information on the choice of priors and Markov chain Monte Carlo settings
☒	☐	For hierarchical and complex designs, identification of the appropriate level for tests and full reporting of outcomes
☐	☒	Estimates of effect sizes (e.g. Cohen's *d*, Pearson's *r*), indicating how they were calculated

Our web collection on statistics for biologists contains articles on many of the points above.

Software and code

Policy information about availability of computer code

Data collection	Prism8: GraphPad https://www.graphpad.com/scientific-software/prism/<br>iBright Analysis Software Thermo Scientific: https://www.thermofisher.com/us/en/home/life-science/protein-biology/protein-assays-analysis/western-blotting/detect-proteins-western-blot/western-blot-imaging-analysis/ibright-western-blot-imaging-systems/ibright-analysis-software-connectivity.html<br>StepOne and StepOnePlus Software v2.3 Applied Biosystems: https://www.thermofisher.com/us/en/home/technical-resources/software-downloads/StepOne-and-StepOnePlus-Real-Time-PCR-System.html<br>EVOS M5000 Imaging System Software Invitrogen: https://www.thermofisher.com/us/en/home/technical-resources/software-downloads/evos-m5000-imaging-system-software-download.html<br>Capillary liquid chromatography interfaced to a mass spectrometer (nano-LC-MS/MS).
Data analysis	ImageJ National Institutes of Health: https://imagej.nih.gov/ij/<br>Adobe Photoshop Version 24.x Adobe: https://www.adobe.com/products/photoshop.html<br>Adobe Illustrator Version 26.4 Adobe: https://www.adobe.com/products/illustrator.html<br>Chip-seq Analysis Software R/Bioconductor package and MACS peak-finding software<br>WashU Epigenome Browser, GSEA analysis<br><br>Mass Spectrometry<br>Post 48hr of transfection, cells were processed for LCMS/MS analysis. Samples were digested overnight with modified sequencing grade trypsin (Promega, Madison, WI), Glu-C (Worthington, Lakewood, NJ), or Arg-C (Roche,Switzerland). Phosphopeptides were enriched using Phospho Select IMAC resins (Sigma). A nanoflow ultra high performance liquid chromatograph (RSLC, Dionex, Sunnyvale, CA) coupled to an electrospray bench top orbitrap mass spectrometer (Q-Exactive plus, Thermo, San Jose, CA) was used for tandem mass spectrometry peptide

sequencing experiments. The sample was first loaded onto a pre-column (2 cm x 100 µm ID packed with ClS reversedphase resin, 5 µm, lO0A) and washed for 8 minutes with aqueous 2% acetonitrile and 0.04% trifluoroacetic acid. The trapped peptides were eluted onto the analytical column, (ClS, 75 µm ID x 50 cm, 2 µm, 100A, Dionex, Sunnyvale, CA). The 90-minute gradient was programmed as: 95% solvent A (2% acetonitrile + 0.1% formic acid) for 8 minutes, solvent B (90% acetonitrile + 0.1% formic acid) from 5% to 38.5% in 60 minutes, then solvent B from 50% to 90% Bin 7 minutes and held at 90% for 5 minutes, followed by solvent B from 90% to 5% in 1 minute and re-equilibrate for 10 min. The flow rate on analytical column was 300 nl/min. Sixteen tandem mass spectra were collected in a data-dependent manner following each survey scan. Both MS and MS/MS scans were performed in Orbitrap to obtain accurate mass measurement using 60 second exclusion for previously sampled peptide peaks. Sequences were assigned using Sequest (Thermo) and Mascot (www.matrixscience.com) database searches against SwissProt protein entries of the appropriate species. Oxidized methionine, carbamidomethyl cysteine, and phosphorylated serine, threonine and tyrosine were selected as variable modifications, and as many as 3 missed cleavages were allowed. The precursor mass tolerance was 20 ppm and MS/MS mass tolerance was 0.05 Da. Assignments were manually verified by inspection of the tandem mass spectra and coalesced into Scaffold reports (www.proteomesoftware.com).

Quantitative RT-PCR analyses were performed using ABI PRISM 7900HT Sequence Detection System (Applied Biosystems). Data were analyzed using StepOne and StepOnePlus software version 2.3 and exported into an Excel spreadsheet. The actin or 18s rRNA data were used for normalizing the gene values, i.e., ng gene/ng actin or 18s rRNA per well. Images were captured using an EVOS M5000 microscope (Invitrogen) and analyzed with ImageJ. All the figures were compiled using Adobe Photoshop Version 24.x and Adobe Illustrator Version 26.4. Percent cell viability was calculated by normalizing all the readings at each inhibitor concentration to the readings exhibited by cultures treated with vehicle alone (i.e., DMSO). The half-maximal inhibitory concentrations (IC50s) were calculated using GraphPad Prism. Protein quantitation was done by Bradford protein assay (Bio-Rad).

Global lipidomic analysis
Lipid extraction from cells, chromatographic separation technique, and analysis were performed. Briefly, lipids were extracted by the Bligh-Dyer method using water: methanol: dichloromethane (2:2:2) after spiking with internal standards pertaining to lipid classes. The organic layer was dried and suspended in Acetonitrile: water: isopropyl alcohol (10:5:85 with 10mM Ammonium Acetate. Chromatographic separation was achieved by reverse-phase LC/MS and a Shimadzu CTO-20A Nexera X2 UHPLC system including a degasser, binary pump, and a column oven 58. A 1.8um particle 50x2.1mm Acquity HSS UPLC T3 column (Waters, Milford, MA, USA) was used to separate lipids. A TripleTOF 5600 with Turbo VTM ion source (AB Sciex, Concorn, ON, Canada) was used for data acquisition in positive and negative ionization modes. MS2 spectra were acquired using the data-dependent acquisition (DDA) function of the Analyst TF software (AB Sciex, Concord, ON, Canada) with dynamic exclusion for coverage depth. Missing values in the data were imputed using the K nearest-neighbor method (KNN). Data was log2 transformed and internal standard normalized (negative method-15:0-18:1(d7) PE (ISTD) and positive method -18:1(d7) Lyso PC (ISTD)) respectively.
For Figure S8 (comparing Abiraterone-treated vs. untreated), differential lipids were determined by t-test ($p < 0.05$), followed by the Benjamini–Hochberg (BH) procedure for false discovery rate correction (FDR < 0.25). BH was used to control the probability of a Type I error rate due to the testing of multiple hypotheses. For Figure 8 (comparing VCaP and C42 untreated vs Abiraterone-treated cells), one-way ANOVA method was performed ($p < 0.05$) followed by the Benjamini–Hochberg (BH) procedure for false discovery rate correction (FDR < 0.25).

For manuscripts utilizing custom algorithms or software that are central to the research but not yet described in published literature, software must be made available to editors and reviewers. We strongly encourage code deposition in a community repository (e.g. GitHub). See the Nature Portfolio guidelines for submitting code & software for further information.

Data

Policy information about availability of data

All manuscripts must include a data availability statement. This statement should provide the following information, where applicable:

- Accession codes, unique identifiers, or web links for publicly available datasets
- A description of any restrictions on data availability
- For clinical datasets or third party data, please ensure that the statement adheres to our policy

The normalized data for this study have been deposited to the Metabolomics Workbench (https://www.metabolomicsworkbench.org/) under the studyID 3296.

The mass spec data is submitted in ProteomeXchange dataset PXD036894
https://proteomecentral.proteomexchange.org/cgi/GetDataset?ID=PXD036894

ChIP-sequencing datasets generated in this study can be found at
https://www.ncbi.nlm.nih.gov/geo/query/acc.cgi?acc=GSE206856

Source data are provided with this paper. The remaining data are available within the Article, Supplementary Information or Source Data file.

Human research participants

Policy information about studies involving human research participants and Sex and Gender in Research.

Reporting on sex and gender	N/A
Population characteristics	N/A
Recruitment	N/A
Ethics oversight	N/A

Note that full information on the approval of the study protocol must also be provided in the manuscript.

Field-specific reporting

Please select the one below that is the best fit for your research. If you are not sure, read the appropriate sections before making your selection.

☒ Life sciences ☐ Behavioural & social sciences ☐ Ecological, evolutionary & environmental sciences

For a reference copy of the document with all sections, see nature.com/documents/nr-reporting-summary-flat.pdf

Life sciences study design

All studies must disclose on these points even when the disclosure is negative.

Sample size	The number of samples for each assay was indicated in each figure legend. For in vitro assays, the sample sizes (at least three biological replicates) were chosen with these assays yielding statistically significant difference between experimental positive and negative controls and on similar sample sizes. Results are representatives of at least three biological replicates and at least two independent experiments. For in vivo assays, at least 3 mice were chosen for each condition, this sample size was determined by using power calculation for a t-test difference between two or three independent means based on a normally distributed population with equal variance.
Data exclusions	No data was excluded from the analysis.
Replication	Each experiment was repeated atleast twice, thrice in most cases for reproducibility. All primers were tested by gel electrophoresis and melt curves evaluated before using them in the quantitative assays for reproducibility.<br>Western blots were repeated atleast twice to ensure reproducibility and captured digitally using iBright imager. All data is available for review. Replicated experiments were successful and support conclusions drawn in this report.
Randomization	Animals were randomly assigned to two or more groups prior to the injection of cells or drug. The rest of the experiments were not randomized, but independent replicates were often performed in different formats, as mitigation measures to cancel out experimental bias.
Blinding	Investigators were not blinded for any of the experiments, including to the animal assignments for tumor formation studies and molecular analysis as treatment conditions were evident from the data. All tissue sections were independently evaluated by pathologist for presence of antibody validation studies and for tumor versus normal determination.

Reporting for specific materials, systems and methods

We require information from authors about some types of materials, experimental systems and methods used in many studies. Here, indicate whether each material, system or method listed is relevant to your study. If you are not sure if a list item applies to your research, read the appropriate section before selecting a response.

Materials & experimental systems

n/a	Involved in the study	
☐	☒	Antibodies
☐	☒	Eukaryotic cell lines
☒	☐	Palaeontology and archaeology
☐	☒	Animals and other organisms
☒	☐	Clinical data
☒	☐	Dual use research of concern

Methods

n/a	Involved in the study	
☐	☒	ChIP-seq
☐	☒	Flow cytometry
☒	☐	MRI-based neuroimaging

Antibodies

Antibodies used	Anti-FLAG Cell Signaling Tech Cat#14793S Clone D6W5B Lot#5 Dilution 1:1000<br>Anti-HA Cell Signaling Technology, Cat# 2367S Clone 6E2 Lot#5 Dilution 1:1000<br>Anti-MYC Cell Signaling Tech Cat#2276S Clone 9B11 Lot#24 Dilution 1:1000<br>Anti-pTyr Santa Cruz Bio Cat#sc-508 Clone PY20 Lot#D0319 Dilution 1:500<br>Anti-Actin Sigma Cat#A2228 Clone AC-74 Dilution1:9,000<br>Anti-SREBF1 Santa Cruz Bio Cat#sc-365513 Clone A4 Lot#1821 Dilution 1:1000<br>Anti-H2A Cell Signaling Tech Cat#3636S Clone L88A6 Dilution 1:1000<br>Anti-KAT2A/GCN5 Santa Cruz Bio Cat#sc-365321 Clone A11 Lot#D1520 Dilution 1:1000<br>Anti-AR Santa Cruz Bio Cat#sc-7305 Clone 441 Lot#D0921 Dilution 1:1000<br>Fixable Aqua Dead Cell stain Thermo Fisher Scientific, Cat#L34957 Dilution 1:400<br>PE/Cyanine7 anti-mouse CD3ε Biolegend Cat#100220 Clone 17A2 Lot#B277057 Dilution 1:400<br>APC Rat Anti-Mouse CD8a BD Pharmingen Cat#553035 Clone 53-6.7 Lot#1299998 Dilution 1:400

	FITC Rat Anti-mouse PD-1 Biolegend Cat#135213 Clone 29F.1A12 Lot#B277057 Dilution 1:400<br>PerCP/Cyanine5.5 anti-mouse CD223 (LAG-3) Cat#125212 Clone C9B7W Lot#B290991 Dilution 1:400
Validation	The effectiveness of the antibodies was confirmed by performing immunoblotting experiments using the manufacturer's data associated with antibody, and their authentication data.<br>Anti-FLAG (https://www.cellsignal.com/products/primary-antibodies/dykddddk-tag-d6w5b-rabbit-mab-binds-to-same-epitope-as-sigma-s-anti-flag-m2-antibody/14793)<br>Anti-HA (https://www.cellsignal.com/products/primary-antibodies/ha-tag-6e2-mouse-mab/2367)<br>Anti-MYC (https://www.cellsignal.com/products/primary-antibodies/myc-tag-9b11-mouse-mab/2276)<br>Anti-pTyr (https://www.scbt.com/p/p-tyr-antibody-py20)<br>Anti-Actin (https://www.sigmaaldrich.com/US/en/product/sigma/a2228)<br>Anti-SREBF1 (https://www.scbt.com/p/srebp-1-antibody-a-4)<br>Anti-H2A (https://www.cellsignal.com/products/primary-antibodies/histone-h2a-l88a6-mouse-mab/3636)<br>Anti-KAT2A/GCN5 (https://www.scbt.com/p/gcn5-antibody-a-11?gclid=EAIaIQobChMIqLLg1YnB_glVZSyzAB3xSAd-EAAYASAAEgKvs_D_BwE)<br>Anti-AR (https://www.scbt.com/p/ar-antibody-441)<br>Fixable Aqua Dead Cell stain (https://www.thermofisher.com/order/catalog/product/L34957)<br>PE/Cyanine7 anti-mouse CD3ε (https://www.biolegend.com/en-us/products/pe-cyanine7-anti-mouse-cd3-antibody-6060?GroupID=BLG242)<br>APC Rat Anti-Mouse CD8a (https://www.bdbiosciences.com/en-us/products/reagents/flow-cytometry-reagents/research-reagents/single-color-antibodies-ruo/apc-rat-anti-mouse-cd8a.553035)<br>FITC Rat Anti-mouse PD-1 (https://www.biolegend.com/en-us/products/fitc-anti-mouse-cd279-pd-1-antibody-7004?GroupID=BLG7930)<br>PerCP/Cyanine5.5 anti-mouse CD223 (LAG-3) (https://www.biolegend.com/en-us/products/percp-cyanine5-5-anti-mouse-cd223-lag-3-antibody-8141?GroupID=BLG5408)

Eukaryotic cell lines

Policy information about cell lines and Sex and Gender in Research

Cell line source(s)	VCaP, HEK293T, C4-2B, TRAMP-C2 cells were obtained from ATCC.
Authentication	Identities of all cell lines were confirmed by Short Tandem Repeat (STR) Profiling.
Mycoplasma contamination	All cultures were tested for mycoplasma contamination every 2 months using the PCR Mycoplasma Test Kit I/C (PromoKine). Mycoplasma testing was negative.
Commonly misidentified lines<br>(See ICLAC register)	None

Animals and other research organisms

Policy information about studies involving animals; ARRIVE guidelines recommended for reporting animal research, and Sex and Gender in Research

Laboratory animals	5-6-week-old male C57BL/6 mice and SCID mice were purchased from Charles River Laboratories. Number mice used per experiments are described in manuscript.
Wild animals	No wild animals were used in the study.
Reporting on sex	Male mice were used in this study.
Field-collected samples	No wild animals were used in the study.
Ethics oversight	All animal studies were performed under approved Institutional Animal Care and Use Committee protocols at Washington University in St. Louis; Approved IACUC protocol # 20180259

Note that full information on the approval of the study protocol must also be provided in the manuscript.

ChIP-seq

Data deposition

☒ Confirm that both raw and final processed data have been deposited in a public database such as GEO.

☒ Confirm that you have deposited or provided access to graph files (e.g. BED files) for the called peaks.

Data access links<br>*May remain private before publication.*	GEO Accession GSE206856 (https://www.ncbi.nlm.nih.gov/geo/query/acc.cgi?acc=GSE206856).
Files in database submission	VCaP Un

Files in database submission	VCaP Romi<br>VCaP Input<br>C4-2B AC130_abi<br>C4-2B AC130_romi
Genome browser session (e.g. UCSC)	No longer applicable

Methodology

Replicates	Each sample was run in duplicate
Sequencing depth	Ten nanograms of immunoprecipitated DNA from VCaP and C4-2B cell lines was used to generate sequencing libraries using the Kapa Hyper Prep Kit (Roche Sequencing Solutions Inc., Pleasanton, CA). The size and quality of the library was evaluated using the Agilent BioAnalyzer (Agilent Technologies, Inc., Santa Clara, CA), and the library was quantitated with the Kapa Library Quantification Kit. Each enriched DNA library was then sequenced on an Illumina NextSeq 500 sequencer to generate 40-50 million 75-base paired-end reads (Illumina, Inc., San Diego, CA).
Antibodies	H2A-K130ac antibodies were custom synthesized by 21st Century Biochem, MA.<br>EZview™ Red Anti-HA Affinity Gel MilliporeSigma Cat#E6779
Peak calling parameters	Peak regions were called using the MACS2 software with the following options-f BAMPE-SPMR-q 0.01-broad. he “BAMPE” option was used for calculating fragment lengths from the paired end reads, “SPMR” for normalizing read depths to number of fragments per million reads, “broad” for compositing broad regions from nearby peak regions. The vehicle treated sample was used as the control.
Data quality	The q value (FDR) cutoff was set to 0.01. A total of 1435 peaks were identified in the Romidepsin treated VCaP samples. These peaks were primarily used for further validation using qPCR.
Software	The raw sequence data were aligned using BowTie 2 and binding sites were identified using the MACS peak-finding software

Flow Cytometry

Plots

Confirm that:

☒ The axis labels state the marker and fluorochrome used (e.g. CD4-FITC).

☒ The axis scales are clearly visible. Include numbers along axes only for bottom left plot of group (a 'group' is an analysis of identical markers).

☒ All plots are contour plots with outliers or pseudocolor plots.

☒ A numerical value for number of cells or percentage (with statistics) is provided.

Methodology

Sample preparation	At the end of the study, all mice were humanely euthanized, tumors were extracted and weighed. The tumor draining lymph nodes were collected and single cell suspensions were made. For analysis of T-cell exhaustion, 1x106 cells were stained with anti-CD3 PECy7, anti CD8 APC, anti-PD1 FITC, anti-Lag3 Percpcy5.5 antibodies.
Instrument	Samples were analyzed using BD FACSCanto II (BD Biosciences)
Software	Post-acquisition analysis was done using FlowJo software (Tree Star Inc)
Cell population abundance	The isolated cells from the lymph node almost had 80-85% of Live cells with 75% of CD3 positive cells
Gating strategy	The first gate of forward and side scatter (FSC and SSC) was created. Following this, CD3 gating was performed to identify the T cells. The CD3 by CD4/CD8 gate allowed us to identify CD8+ or CD4+ T cells. The CD8 by PD1/Lag-3 gating was done to check the population of exhausted T cells.

☒ Tick this box to confirm that a figure exemplifying the gating strategy is provided in the Supplementary Information.
